# Supplementary material for: Repeatome Analysis of Plasma Circulating DNA in Patients with Cardiovascular Disease: Variation with Cell-Free DNA Integrity/Length and Clinical Parameters
Source: Int J Mol Sci. 2025 Jul 11;26(14):6657. doi: 10.3390/ijms26146657 (PMC12294208; doi:10.3390/ijms26146657)
Supplement: Supplementary file 1 [file ijms-26-06657-s001.zip › Supplementary Figure S2 (10-07-2024).pdf]

Figure S2

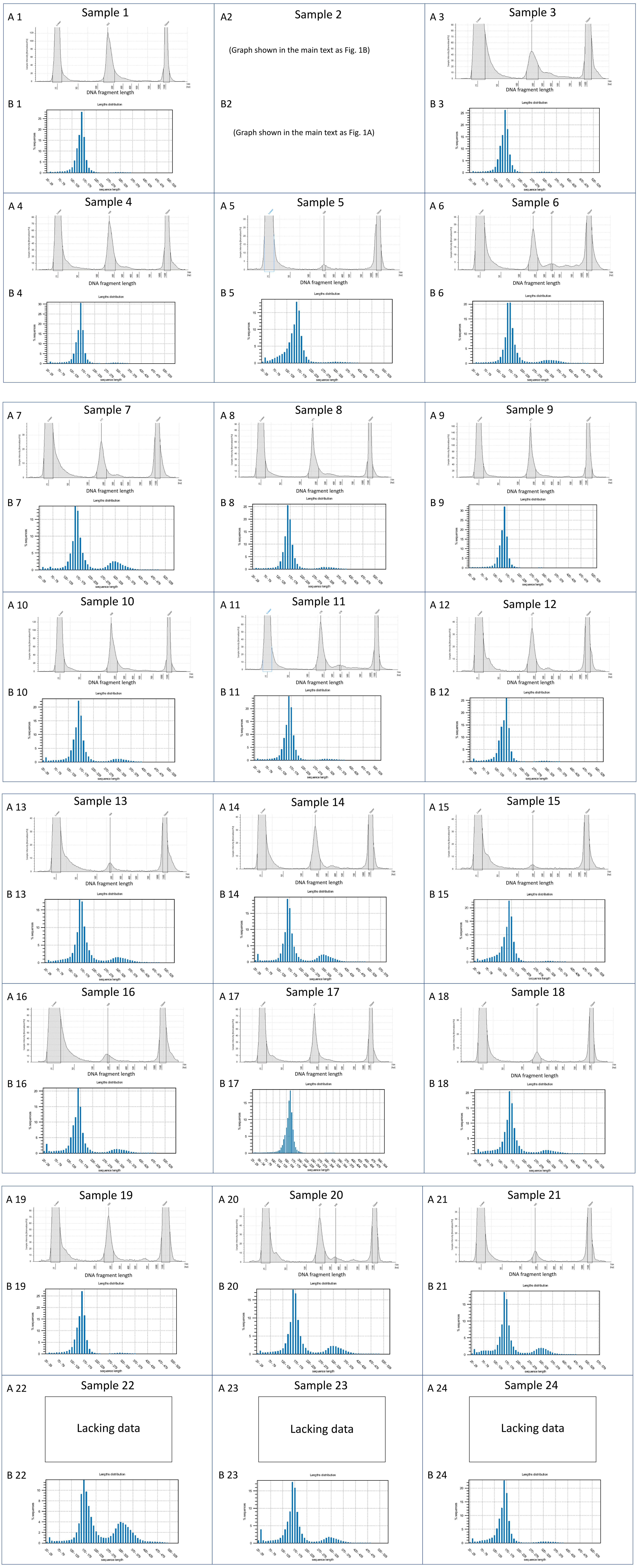

Figure S2. Graphical representation of the length distribution of cfDNA samples. For each of the analysed DNA samples the upper part (A1-A24) of each figure section represents the length distribution of cfDNA analyzed using automated electrophoresis with high-sensitivity D1000 ScreenTapes on the TapeStation 4200 System, while the lower part (B1-B24) represents the length distribution of the reads obtained by next-generation sequencing (NGS). The two peaks around 100-200 bp (always present) and around 300-400 bp (visible in part of the samples) represent the abundance of mono- and di-nucleosomal cfDNA fragments.
