# Supplementary material for: Repeatome Analysis of Plasma Circulating DNA in Patients with Cardiovascular Disease: Variation with Cell-Free DNA Integrity/Length and Clinical Parameters
Source: Int J Mol Sci. 2025 Jul 11;26(14):6657. doi: 10.3390/ijms26146657 (PMC12294208; doi:10.3390/ijms26146657)
Supplement: Supplementary file 1 [file ijms-26-06657-s001.zip › Supplementary Figures S1, S3, S4, S5 (10-07-25).pdf]

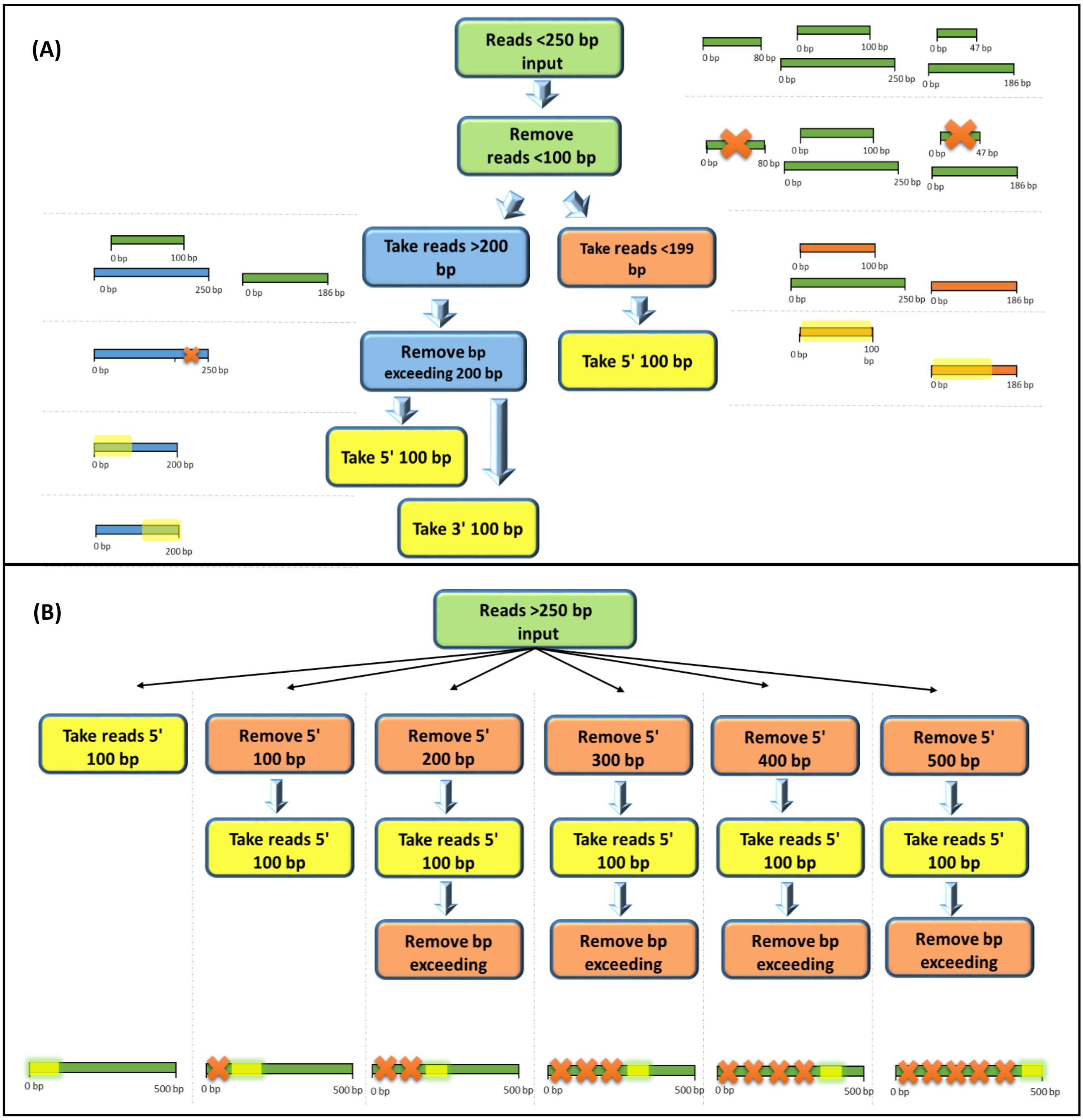

Figure S1. Automated workflow to extract 100 bp fragments from each sequencing read. Step-by-step instructions for extracted 100 bp fragments from (A) mono- and (B) di-nucleosomal sequencing reads (reads <250 bp and >250 bp, respectively) through an automated workflow created with CLC Genomics Workbench version 7.0 software (Qiagen). The textual and graphic description outlines the different stages of the automated process.

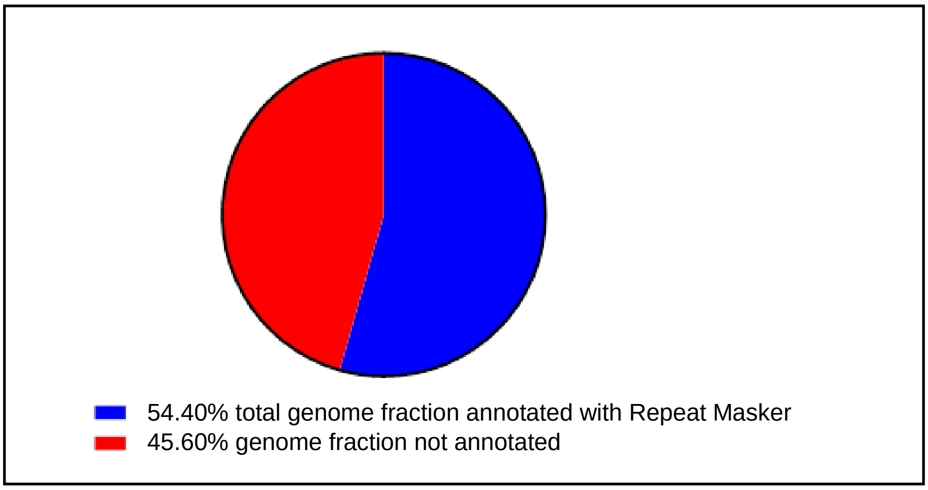

Figure S3. Representation of repetitive and non-repetitive elements in GRCh38 annotated with Repeat Masker. The pie charts illustrate the overall percentage distribution of genome fraction annotated with Repeat Masker and not.

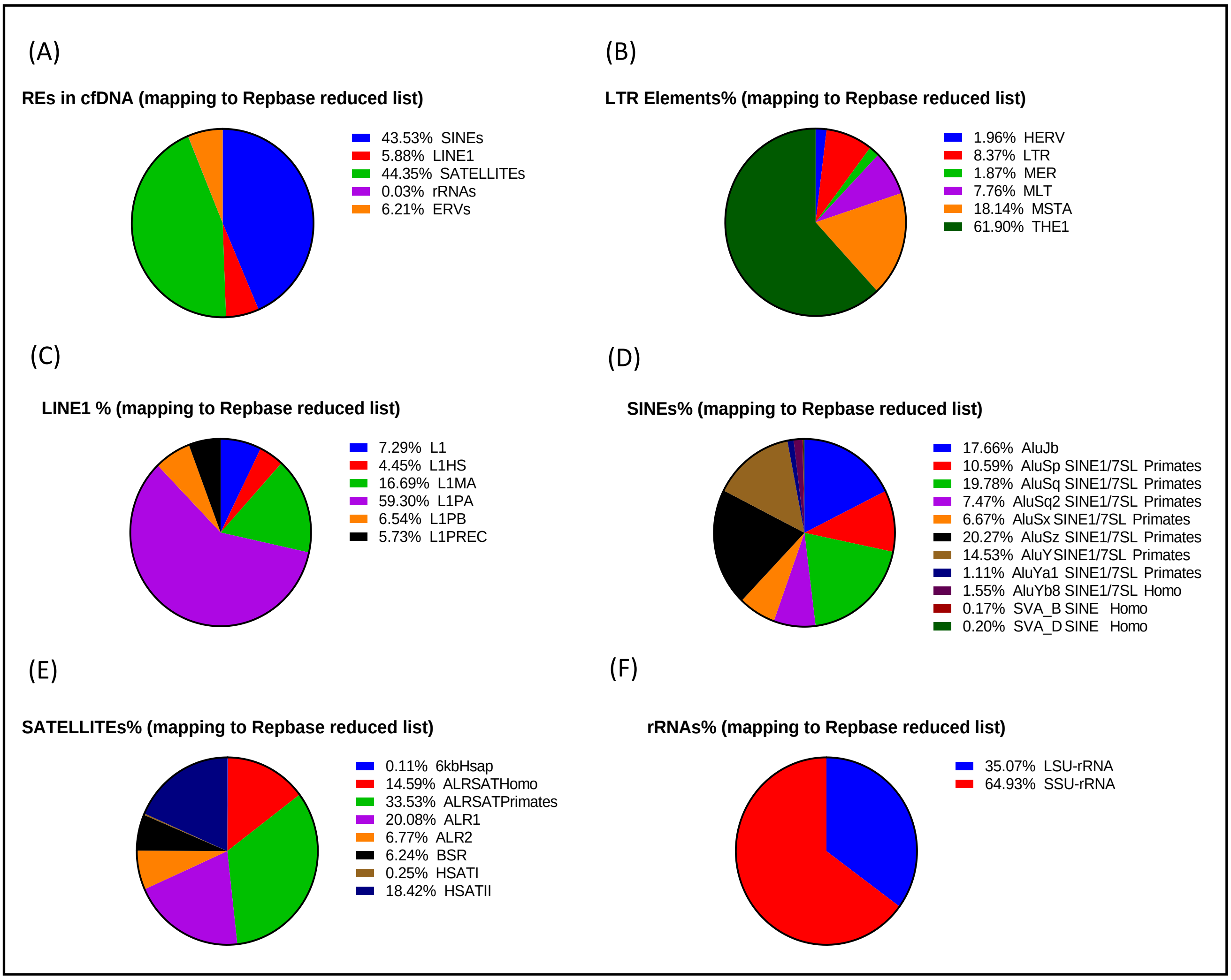

Figure S4. Identification of significantly overrepresented repetitive elements in cell-free DNA. The analysis was performed aligning cfDNA 100 bp fragment reads to “Repbase reduced list” of repetitive elements consensus sequences. The pie chart (A) illustrates the overall distribution of cfDNA 100 bp fragment sequences among the main families of repetitive elements; the other graphics illustrate the representation of the subfamilies that constitute each of the repetitive element groups captions mentioned, including (B) LTR elements, (C) LINE1, (D) SINEs, (E) SATELLITEs and (F) rRNAs.

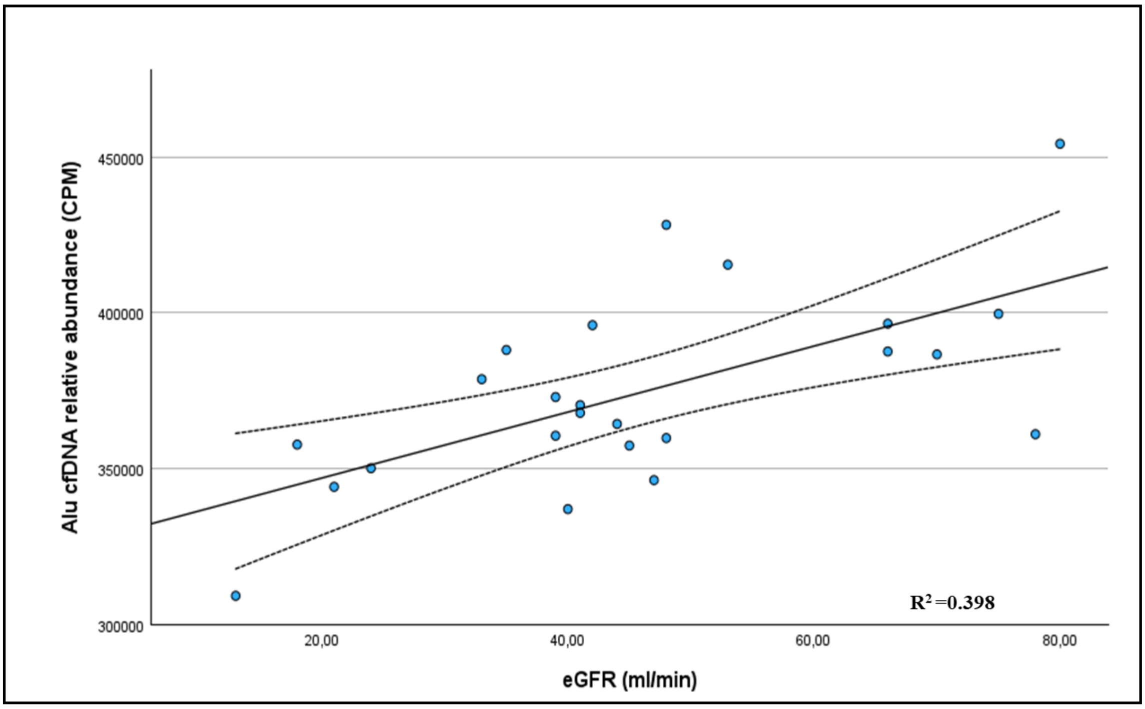

Figure S5. Relationship between eGFR and Alu count per million in cfDNA >250 bp. Adaptation curve with 95% C.I. is shown.
