# Supplementary material for: Repeatome Analysis of Plasma Circulating DNA in Patients with Cardiovascular Disease: Variation with Cell-Free DNA Integrity/Length and Clinical Parameters
Source: Int J Mol Sci. 2025 Jul 11;26(14):6657. doi: 10.3390/ijms26146657 (PMC12294208; doi:10.3390/ijms26146657)
Supplement: Supplementary file 1 [file ijms-26-06657-s001.zip › Supplementary Tables (10-07-2025).pdf]

Table S1. “*Repbase reduced list*”.

Reference sequence	Reference length	Contig mapped	Average Coverage*	Repeat classes
**L1 L1 Homo**	5403	32	2.327041	L1 (Other)
**L1HS L1 Homo**	6064	13	0.730706	L1HS
**L1PREC1 L1 Homo**	6460	13	0.734056	L1P
**ALR SAT Homo**	171	10	9.877193	Alpha satellite
**L1PA3 L1 Homo**	902	10	3.370288	L1P
**L1PA7 L1 Homo**	901	10	4.039956	L1P
**THE1_I LTR**	1580	10	2.197468	ERV (Other)
**ALR1 SAT Homo**	171	7	6.760234	Alpha satellite
**L1PA4 L1 Homo**	902	7	1.949002	L1P
**L1PA7_5 L1 Homo**	1727	7	1.624783	L1P
**AluY SINE1/7SL Primates**	282	6	6	AluY
**L1PA13 L1 Homo**	910	5	2.102198	L1P
**L1PA5 L1 Homo**	901	5	1.400666	L1P
**L1PA6 L1 Homo**	901	5	1.866815	L1P
**LTR12C ERV1 Homo**	1577	5	1.257451	HERV-LTR
**AluSx SINE1/7SL Primates**	283	4	3.982332	AluS
**L1PB2c L1 Homo**	6582	4	0.231237	L1P
**6kbHsap satellite Homo**	6018	3	0.387172	Beta/acrocentric satellites
**ALR_ SAT Primates**	171	3	2.614035	Alpha satellite
**AluSq SINE1/7SL Primates**	284	3	3	AluS
**L1PA8 L1 Homo**	919	3	0.603917	L1P
**L1PREC2 L1 Homo**	8145	3	0.198772	L1P
**LSU-rRNA_Hsa rRNA Metazoa**	5035	3	0.726117	rRNA
**HERVH ERV1 Eutheria**	7713	2	0.190976	HERV
**L1PB1 L1 Homo**	898	2	0.888641	L1P
**LTR7A ERV3 Homo**	450	2	0.951111	HERV-LTR
**MSTA ERV3 Primates**	428	2	1.995327	ERV (Other)
**SVA_D SINE Homo**	1386	2	0.87518	SVA
**THE1B ERV3 Hominidae**	364	2	1.629121	ERV (Other)
**THE1C ERV3 Homo**	375	2	1.338667	ERV (Other)
**THE1D ERV3 Homo**	381	2	1.587927	ERV (Other)
**ALR2 SAT Homo**	179	1	0.960894	Alpha satellite
**AluJb SINE1/7SL Primates**	283	1	0.918728	AluJ
**AluSp SINE1/7SL Primates**	284	1	1	AluS
**AluSq2 SINE1/7SL Primates**	286	1	1	AluS
**AluSz SINE1/7SL Primates**	283	1	1	AluS
**AluYa1 SINE1/7SL Primates**	282	1	1	AluY
**AluYb8 SINE1/7SL Homo**	289	1	1	AluY
**BSR SAT Homo**	136	1	1	Beta/acrocentric satellites
**HERV9 ERV1 Homo**	8399	1	0.062746	HERV
**HERVK11DI ERV2 Homo**	7752	1	0.282895	HERV

**HERVL ERV3 Homo**	5654	1	0.074637	HERV
**HSATI SAT Primates**	577	1	0.989601	Human satellites
**HSATII SAT Primates**	170	1	0.905882	Human satellites
**L1MA2 L1 Homo**	1051	1	0.195052	L1 (Other)
**L1MA9 L1 Homo**	1059	1	0.167139	L1 (Other)
**L1PA10 L1 Homo**	915	1	0.215301	L1P
**L1PA14 L1 Homo**	908	1	0.431718	L1P
**L1PA15 L1 Homo**	912	1	0.319079	L1P
**L1PA16 L1 Homo**	913	1	0.508215	L1P
**L1PA16_5 L1 Homo**	4083	1	0.161891	L1P
**L1PA2 L1 Homo**	902	1	0.189579	L1P
**LTR12 ERV1 Primates**	826	1	0.478208	HERV-LTR
**LTR12E ERV1 Homo**	1322	1	0.217852	HERV-LTR
**LTR5_Hs ERV2 Primates**	968	1	0.713843	HERV-LTR
**MER11C ERV2 Homo**	1071	1	0.45098	ERV (Other)
**MLT2A1 ERV3 Homo**	444	1	0.585586	ERV (Other)
**MLT2A2 ERV3 Homo**	549	1	0.260474	ERV (Other)
**SSU-rRNA_Hsa rRNA Metazoa**	1869	1	1	rRNA
**SVA_B SINE Homo**	1383	1	0.229212	SVA
**THE1A ERV3 Homo**	355	1	0.833803	ERV (Other)

*Note. * Coverage referred to the mapping of the contig's consensus sequences versus the RepBase full list consensus sequence.*

Table S2. Count per million (CPM) of 100 bp sequences corresponding to different classes of repetitive DNA elements, in mono- and di-nucleosomal cfDNA fragments

**Repetitive DNA family**	**Mono-nucleosomal cfDNA (< 250 bp) (Mean +- sd)**	**Di-nucleosomal cfDNA (> 250 bp) (Mean +- sd)**	**P-value[(u)]**	**P-value[(a)]**
**rRNA**	1754 ± 410	4417 ± 3107	<0.001	<0.01
**Alpha satellites**	161729 ± 12483	129164 ± 13488	<0.001	<0.001
**Beta/acrocentric satellites**	17500 ± 1333	20688 ± 3260	<0.001	<0.001
**Human satellites**	38885 ± 6670	41298 ± 7920	<0.05	n.s.
**SVA**	5779 ± 533	6943 ± 1328	<0.001	<0.01
**Alu-J**	57247 ± 2289	65155 ± 5155	<0.001	<0.001
**Alu-S**	209359 ± 7866	243295 ± 23819	<0.001	<0.001
**Alu-Y**	55080 ± 2217	66110 ± 6394	<0.001	<0.001
**HERV**	21863 ± 894	22885 ± 2114	<0.05	n.s.
**HERV-LTR**	13360 ± 839	17344 ± 1824	<0.001	<0.001
**Other ERV**	78258 ± 2863	85281 ± 5732	<0.001	<0.001
**L1HS**	43495 ± 1536	40505 ± 5445	<0.05	n.s.
**L1P**	202173 ± 8364	175795 ± 9774	<0.001	<0.001
**L1 (Other)**	93095 ± 3793	82088 ± 7236	<0.001	<0.001

Note: [(u)] = Paired sample T test P-value, unadjusted; [(a)]= Paired sample T test P-value, Bonferroni adjusted considering the multiple testing for 14 repeat classes

Table S3-A. Description of clinical and laboratory characteristics of the patients.

	Mean (s.d.)	Min.-max.	Percentage	N° of cases
Patients' characteristics				
**Age**, (yrs) mean (s.d.)	87.31 (4.63)	80.09-96.07		24
**Survival**, Time to event (months)	13.58 (10.73)	0-24		24
**Survival**, categorical				
1-24 months			(50%)	12
> 24 months			(50%)	12
**Sex**, n (%)				
Female			(41.7%)	10
Male			(58.3%)	14
Diagnosis at admission				
**Hearth failure, n (%)**			100%	24
*Comorbidities**				
**Chronic kidney disease (CKD)**			45.83%	11
**Hypertension**			87.5%	21
**CAD/PAD**			33.33 %	8
**Cardiac arrhytmias**			41.66 %	10
**Diabetes/dyslipidemia**			37.5 %	9
**Anemia**			33.33 %	8
Routine laboratory parameters				
	**Mean (s.d.)**	**Min.-max.**	**%**	**N° of cases**
**WBC x10^3/µl**	10.32 (5.30)	5.07-29.01		24
**RBC x10^6/µl**	3.90 (0.60)	2.96-4.94		24
**Neutrophils x10^3/µl**	8.25 (5.01)	3.20-25.82		24
**NLR**	8.58 (8.09)	1.81-31.81		24
**Creatinine, (mg/dL)**	1.42 (0.58)	0.70-2.90		24
**eGFR, (ml/min)**	46.08 (18.57)	13.00-80.00		24
**Troponin, (pg/ml)**	54.69 (43.37)	7.24-215.70		20
**Nt-proBNP, (pg/ml)**	7345.75 (7474.44)	247-26438		16
**CRP, (mg/dl)**	7.29 (7.33)	0.11-18.63		15
cfDNA parameters by Real-time PCR (Alu DNA quantification)				
	**mean (s.d.)**	**Min.-max.**	**%**	**N° of cases**
**Alu 115 ng/ml**	46.14 (48.07)	2.75-206.75		24
**Alu 247 ng/ml**	9.70 (8.47)	1.00-32.75		24
**Alu 247/115**	0.26 (0.14)	0.10-0.76		24

Alu 247/115 (categorical)		
(tertile 1) Low integrity	(45.8%)	11
(tertile 2) Intermediate integrity	(29.2%)	7
(tertile 3) High integrity	(25%)	6

Other cfDNA parameters (Agilent Tape Station Screen Tape Analysis)				
	**mean (s.d.)**	**Min.-max.**	**%**	**N° of cases**
**Plasma concentration of mono-nucleosomal cfDNA by H.S. screen tape (range 100-280bp), pmol/L**	593.60 (458.60)	60.40-1690.00		21
**Plasma concentration of di-nucleosomal and tri-nucleosomal cfDNA by H.S. screen tape (range 280-700 bp), pmol/L**	48.70 (31.57)	11.90-99.20		21
**cfDNA_integrity by H.S. screen tape (pmol/L range 280-700bp)/ (pmol/L range 100-280bp)**	0.11 (0.053)	0.04-0.21		21

*Note: * additional (less frequent) comorbidities: Chronic diseases of the digestive system, n=2; Acute diseases of the digestive system, n=4; Acute Diseases of the urinary system, n=2; Degenerative diseases of the CNS, n=4; Bone & Muscle Diseases, n=2; Endocrine, Nutritional and Metabolic Diseases, n=2; COPD, n=5; pneumonia, n= 1; Unspecified pleural Effusion, n=3; acute respiratory failure, n=5*

Table S3-B. Description of clinical and laboratory characteristics of the patients in the two subgroups with different outcome

	**Survived after follow-up**				**Not survived after follow-up**				
	Mean (s.d.)	Min.-max.	Percent age	N° of cases	Mean (s.d.)	Min.-max.	Percent age	N° of cases	p-value
Patients' characteristics									
**Age**, (yrs)	86.65 (5.59)	80.09-96.07		12	87.97 (3.56)	81.07-92.14		12	0.347§
**Survival**, Time to event (months)	24 (0.00)	24-24		12	3.17 (1.99)	0-6		12	
**Sex**, n (%)									
Female			(41.7%)	5			(41.7%)	5	1.00¥
Male			(58.3%)	7			(58.3%)	7	
Comorbidities									
**Chronic kidney disease (CKD)**									
Yes			58.3 %	7			50%	6	1.00¥

No			41.7 %	5			50%	6	
**Hypertension**									
Yes			0 %	0			25%	3	0.217¥
No			100 %	12			75%	9	
**CAD/PAD**									
Yes			58.3 %	7			75%	9	0.667¥
No			41.7 %	5			25%	3	
**Cardiac arrhytmias**									
Yes			75.0 %	9			41.7%	5	0.214¥
No			25.0 %	3			58.3%	7	
**Diabetes/dyslipidemia**									
Yes			50.0 %	6			75 %	9	0.400¥
No			50.0 %	6			25 %	3	
**Anemia**									
Yes			75.0 %	9			58.3 %	7	0.667¥
No			25.0 %	3			41.7 %	5	
Routine laboratory parameters									
**WBC x10^3/µl**	8.02	5.07-14.27		12	12.62	6.00-29.01		12	**0.008**
**RBC x10^6/µl**	3.75	2.96-4.35		12	4.05	3.21-4.94		12	0.219
**Neutrophils x10^3/µl**	6.04	3.20-11.74		12	10.44	4.98-25.82		12	0.713
**NLR**	5.71	1.81-15.21		12	11.45	2.61-31.81		12	0.143
**Creatinine, (mg/dL)**	1.54	0.90-2.90		12	1.30	0.70-2.40		12	0.410
**eGFR, (ml/min)**	42.75	13.00-78.00		12	49.41	18.00-80.00		12	0.514
**Troponin, (pg/ml)**	43.63	7.24-96.50		10	65.75	22.98-215.70		10	0.280
**Nt-proBNP, (pg/ml)**	9185.56	2435-26438		9	4980.29	247-12990		7	0.351
**CRP, (mg/dl)**	6.72	0.11-18.63		8	7.98	1.60-18.58		7	0.336

Note: § Mann-Whitney U test. ¥ Fisher exact test.

Table S4. Correlation (Spearman rho) between cfDNA parameters and parameters of clinical risk

	Age	Time to event (days)	NLR (n=24)	Wbc	Rbc	Creatinine	eGFR (n=24)	Troponin (n=20)	Nt-proBNP (n= 16)	CRP (n=15)
**Dinucleosomal/mononucleosomal cfDNA ratio on H.S. Screen Tape, pg/ul (n=21)**	0.179	-0.051	-0.121	-0.139	0.136	**0.523***	**-0.517***	0.252	0.126	0.473
**Dinucleosomal/mononucleosomal cfDNA in NGS reads (n=24)**	-0.043	0.192	-0.226	-0.086	0.209	0.234	-0.248	-0.163	-0.124	-0.168

*Note: * = p-value < 0.05.*

Table S5. cfDNA composition of repetitive DNA families in patients groups with different survival

Repeat class	Survival (1 = deceased)	Mononucleosomal cfDNA Mean [95% C.I.]	Median (IQR)	P-value$^{(u)}$	Dinucleosomal cfDNA Mean [95% C.I.]	Median (IQR)	P-value$^{(u)}$
**Alu-J**	0	5.72x10^{04} (5.59x10^{04} – 5.84x10^{04})	5.69x10^{04} (3.25x10^{03})	0.799	6.37x10^{04} (6.06x10^{04}-6.68x10^{04})	6.28x10^{04} (4.66x10^{03})	0.128
	1	5.73x10^{04} (5.56x10^{04}-5.90x10^{04})	5.77x10^{04} (4.42x10^{03})		6.66x10^{04} (6.33x10^{04}-6.99x10^{04})	6.54x10^{04} (5.27x10^{03})	
**Alu-S**	0	2.09x10^{05} (2.05x10^{05}-2.14x10^{05})	2.10x10^{05} (6.31x10^{03})	0.932	2.37x10^{05} (2.25x10^{05}-2.49x10^{05})	2.38x10^{05} (2.29x10^{04})	0.443
	1	2.10x10^{05} (2.04x10^{05}-2.15x10^{05})	2.09x10^{05} (1.27x10^{04})		2.50x10^{05} (2.33x10^{05}-2.67x10^{05})	2.49x10^{05} (2.82x10^{04})	
**Alu-Y**	0	5.50x10^{04} (5.34x10^{04}-5.65x10^{04})	5.50x10^{04} (3.17x10^{03})	0.843	6.48x10^{04} (6.12x10^{04}-6.85x10^{04})	6.47x10^{04} (7.57x10^{03})	0.443
	1	5.52x10^{04} (5.39x10^{04}-5.65x10^{04})	5.50x10^{04} (3.23x10^{03})		6.74x10^{04} (6.29x10^{04}-7.18x10^{04})	6.69x10^{04} (1.18x10^{04})	
**Alu (total)**	0	3.21x10^{05} (3.14x10^{05}-3.28x10^{05})	3.23x10^{05} (1.12x10^{04})	1.00	3.65x10^{05} (3.48x10^{05}-3.82x10^{05})	3.61x10^{05} (3.13x10^{04})	0.178
	1	3.22x10^{05} (3.14x10^{05}-3.30x10^{05})	3.19x10^{05} (1.83x10^{04})		3.84x10^{05} (3.63x10^{05}-4.05x10^{05})	3.83x10^{05} (3.95x10^{04})	
**L1HS**	0	4.33x10^{04} (4.22x10^{04}-4.44x10^{04})	4.37x10^{04} (3.23x10^{03})	0.551	3.97x10^{04} (3.77x10^{04}-4.17x10^{04})	4.03x10^{04} (4.47x10^{03})	0.378
	1	4.37x10^{04} (4.28x10^{04}-4.46x10^{04})	4.37x10^{04} (2.01x10^{03})		4.13x10^{04} (3.68x10^{04}-4.58x10^{04})	4.13x10^{04} (4.85x10^{03})	
**ALR**	0	1.64x10^{05} (1.56x10^{05}-1.72x10^{05})	1.66x10^{05} (1.79x10^{04})	0.630	1.29x10^{05} (1.22x10^{05}-1.37x10^{05})	1.30x10^{05} (1.09x10^{04})	0.799
	1	1,60x10^{05} (1,52x10^{05}-1,68x10^{05})	1,60x10^{05} (2,11x10^{04})		1,29x10^{05} (1,19x10^{05}-1,39x10^{05})	1,34x10^{05} (2,06x10^{04})	

Note: $^{(u)}$ = Mann-Whitney, P-value, unadjusted.

Table S6. Fold change of repeat families in cfDNA in patients groups with or without CKD

Repeat class	CKD (1=ckd)	Mean (95% C.I.)	Median (IQR)	P-value$^{(u)}$	P-value$^{(a)}$
**FC_AluJ**	0	1.14 (1.09-1.20)	1.13 (0.08)	0.865	n.s.
	1	1.13 (1.09-1.18)	1.13 (0.10)		
**FC_AluS**	0	1.20 (1.16-1.25)	1.20 (0.08)	**0.006**	**0.03**
	1	1.11 (1.07-1.16)	1.12 (0.08)		
**FC_AluY**	0	1.21 (1.14-1.29)	1.20 (0.19)	0.608	n.s.
	1	1.18 (1.11-1.26)	1.17 (0.15)		
**FCTot_Alu**	0	1.19 (1.15-1.23)	1.19 (0.13)	**0.047**	n.s.
	1	1.13 (1.08-1.17)	1.13 (0.09)		

Note: $^{(u)}$ = Mann-Whitney. P-value. unadjusted; $^{(a)}$= Mann-Whitney. P-value. Bonferroni adjusted considering the multiple testing for 3 categories (AluJ, AluS, AluY)
